# Design of stable magnetic hybrid nanoparticles of Si-entrapped HRP

**DOI:** 10.1371/journal.pone.0214004

**Published:** 2019-04-01

**Authors:** Sonali Correa, Sara Puertas, Lucía Gutiérrez, Laura Asín, Jesús Martínez de la Fuente, Valeria Grazú, Lorena Betancor

**Affiliations:** 1 Laboratorio de Biotecnología, Universidad ORT Uruguay, Montevideo, Uruguay; 2 Nanoimmunotech S.L., Zaragoza, Spain; 3 Instituto de Nanociencia de Aragón, Universidad de Zaragoza, Campus Río Ebro, Edificio I+D, Zaragoza, Spain; 4 Instituto de Ciencia de Materiales de Aragón (ICMA), Consejo Superior de Investigaciones Científica, Zaragoza, Spain; 5 Centro de Investigación Biomédica en Red de Bioingeniería, Biomateriales y Nanomedicina (CIBER-BBN), Madrid, Spain; Queen's University Belfast, UNITED KINGDOM

## Abstract

Hybrid and composite nanoparticles represent an attractive material for enzyme integration due to possible synergic advantages of the structural builders in the properties of the nanobiocatalyst. In this study, we report the synthesis of a new stable hybrid nanobiocatalyst formed by biomimetic silica (Si) nanoparticles entrapping both Horseradish Peroxidase (HRP) (EC 1.11.1.7) and magnetic nanoparticles (MNPs). We have demonstrated that tailoring of the synthetic reagents and post immobilization treatments greatly impacted physical and biocatalytic properties such as an unprecedented ~280 times increase in the half-life time in thermal stability experiments. The optimized nanohybrid biocatalyst that showed superparamagnetic behaviour, was effective in the batch conversion of indole-3-acetic acid, a prodrug used in Direct Enzyme Prodrug Therapy (DEPT). Our system, that was not cytotoxic *per se*, showed enhanced cytotoxic activity in the presence of the prodrug towards HCT-116, a colorectal cancer cell line. The strategy developed proved to be effective in obtaining a stabilized nanobiocatalyst combining three different organic/inorganic materials with potential in DEPT and other biotechnological applications.

## Introduction

The possibilities for practical applications of immobilized enzymes are continuously growing and a steady number of immobilization methods have been recently developed to preserve the activity of biotechnologically important enzymes in unnatural environments [1–4]. Apart from industrial large-scale applications, immobilization techniques on nanoscale supports have enabled and amplified the integration of enzymes in biosensors [5–7], nano/microreactors [8,9] or in the generation of hybrid nanostructures for biomedical applications [8,10,11]. Design of tailored strategies for enzyme immobilization has proved essential to achieve better specific activities, robustness and reusability of the material integrated enzymes, addressing the major problems that restrain industrial or therapeutic implementation of enzymatic reactions [12–14].

Selection of the support material for enzyme immobilization is a critical aspect due to its major impact on the properties of the biocatalyst. Its shape and textural characteristics of, hydrophilicity/hydrophobicity properties, biocompatibility, toxicity or physicochemical stability can directly influence the performance and utility of the immobilized enzyme [15–17]. Consequently, the discovery and use of new support materials with desired properties has become extremely important in the design of immobilized biocatalysts.

In this regard, scientific attention has been directed towards hybrid and composite materials, which combine properties of both composite precursor and maximize their advantages [4,18]. Upon integration of the different materials, it is desired that the intrinsic characteristics of each individual component are preserved, exhibiting new additional properties due to the synergetic effect between the structural builders. When immobilizing an enzyme in composite supports, scientists aim for a combined benefit of the materials on the properties of the biocatalyst.

Biomimetic silica (Si) has been used in the past to generate hybrid inorganic/organic nanocomposites for a range of applications. It can be synthesized as a nanostructured material with divergent morphologies within minutes under mild and green conditions [19,20]. Any material contained in the synthetic mixture may become entrapped within the biomimetic Si nanoparticles [17,21–23]. The mild synthetic approach (room temperature, neutral pH, free of organic solvents) is compatible with a range of enzymes for which the strategy has also resulted in stabilization [24–26]. Moreover, for biomedical applications such as enzyme replacement therapies or direct enzyme prodrug activation, encapsulation of enzymes in a Si nanocarrier could reduce the immunogenicity of the enzyme. However, synthetic strategies for biomimetic Si nanobiocatalysts are not universal as they provide distinct properties to different enzymes and may be tailored to improve a desired attribute [13].

HRP is a heme-containing enzyme that uses hydrogen peroxide as electron acceptor. Its importance in biotechnology is long established as it is involved in a variety of biological processes, it is able to amplify weak signals, it is stable towards external factors (e.g. peroxide species, temperature) and has a high turnover number [27]. In the past decade, HRP related investigations have regained interest following the discovery of new natural isoenzymes with different biochemical properties and the development of an efficient recombinant expression system that facilitated its production. New HRP properties might enable the effective use of this enzyme in polymer synthetic reactions in the presence of organic solvents or as a therapeutic agent in cancer therapy [28,29]. In the light of these new applications, we believe it is timely to propose novel immobilization strategies for HRP on tailored materials that augment its practical possibilities.

In this study, we demonstrate a new approach to integrate and stabilize HRP in biohybrid magnetic nanoparticles (biomimetic Si + magnetic nanoparticles (MNPs)). The hybrid immobilization system provided ease of separation in biocatalytic applications or accumulation of HRP nanobiocatalyst where desired. Each material included in the nano-hybrid contributed to the improvement of the properties of the nanohybrid which enabled its use in the conversion of indole-3- acetic acid (3-IAA), a prodrug used in cancer therapy.

## Materials and methods

Horseradish peroxidase Type VI (EC 1.11.1.7), polyethylenimine (PEI) (MW 1300, 2000, 25000 and 60000), 2,2′-Azino-bis (3-ethylbenzothiazoline-6-sulfonic acid) diammonium salt (ABTS) and hydrogen peroxide were from Sigma Aldrich (St. Louis, MO). Tetramethyl orthosilicate (TMOS), trehalose and potassium phosphate monobasic were from MERCK (Whitehouse Station, NJ). Dibasic sodium phosphate and sodium acetate were from Biopack (Buenos Aires, Argentina). Gel filtration PD10-Columns were from GE Healthcare (Buckimghamshire, UK). Magnetic nanoparticles (MNPs) fluidMag-PAA (200 nm of aggregate size) were from Chemicell (Berlin, Germany). All other chemicals used were analytical grade reagents.

### Determination of HRP activity

The activity of the free and entrapped enzyme preparations was measured by a colorimetric assay using 9.1 mM ABTS, (Ԑ_M_ = 36.8 mM^-1^cm^-1^), as a substrate. The final assay contained 1.7 mL of 0.1 M potassium phosphate, pH 5.0 at 25°C, 0.1 mL of 9.1 mM ABTS, 0.2 mL 0.3% (w/w) hydrogen peroxide solution (H_2_O_2_) in deionized water and 10 μl of the soluble and nanohybrid preparations. The oxidation of ABTS was measured in a spectrophotometer at a wavelength of 405 nm for 2 min (Unico SQ-2800 UV-Vis). One enzyme unit (IU) was defined as the amount of HRP able to oxidize 1 μmol of ABTS in the above-mentioned conditions.

#### Entrapment of HRP in biomimetic Si nanoparticles

Aliquots of 0.4 mL of horseradish peroxidase Type VI solutions (protein concentration varied from 0.5 to 20 mg/mL) in potassium phosphate buffer (0.1 M, pH 8.0) were mixed with 0.1 mL of 10% polyethyleneimine (PEI) adjusted to pH 8.0 with HCl and 0.1 mL of a hydrolyzed TMOS solution prepared by diluting TMOS in hydrochloric acid (1 mM) to a final concentration of 1 M. The enzyme, buffer and PEI were mixed and gently agitated in an end-over-end roller for 15 min at 25°C. Then, the hydrolysed TMOS was added and this mixture was incubated for 5 min at 25°C. The resultant entrapped HRP preparation was then centrifuged (13500 rpm) for 5 min, washed five times by centrifugation and resuspension with sodium phosphate buffer 0.1 M pH 8.0 at 25°C and sonicated in an ultrasonic cleaner at 130 W and 20 kHz, (SONICS & MATERIALS, INC.) for 5 min. The immobilized nanobiocatalysts entrapped in Si are noted as BioSi@HRP.

Immobilization percentage was defined as:

$$\%I=\frac{(Initial\;activity-Activity\;in\;supernatant)*100}{Initial\;activity}$$

Immobilization yield was defined as:

$$\%Y=\frac{(Activity\;in\;immobilized\;preparation)*100}{Initial\;activity-Activity\;in\;supernatant}$$

#### Oxidation of HRP

HRP was oxidized using a modification of Zalipsky’s PEGylation protocol with the aim of generating aldehyde groups in its sugar moieties [30]. Peroxidase (3 mg) was dissolved in 1.8 mL of 10 mM sodium phosphate containing 154 mM sodium chloride, pH 7.2. Simultaneously, 8.6 mg of sodium periodate were dissolved in 200 μL of distilled water and protected from light. The sodium periodate solution was immediately added to the enzyme solution, and the sample was gently agitated. The 2 mL mixture was incubated in the dark for 1 h at 25°C with constant agitation. The reaction was then quenched by the addition of 2.5 μL of glycerol (99.5%) and the oxidized enzyme was then purified by using a desalting PD10 column equilibrated with 100 mM sodium phosphate pH 6.0 containing 154 mM sodium chloride. Oxidized HRP was concentrated to 1 mg/mL using Vivaspin 500 with a 30 KDa cut off membrane. The oxidised enzyme is noted as HRPox.

#### Covalent three-dimensional immobilization of the entrapped HRP

HRPox (1 mg/mL) was entrapped in Si nanoparticles using the above-mentioned protocol. The entrapped enzyme was then incubated in 25 mM sodium bicarbonate, pH 10.0 (R 1:10) overnight at 4°C to facilitate the formation of schiff´s bases between the aldehyde groups generated in the enzyme and unreacted amino group from the support. The shiff´s bases were finally reduced using sodium borohydride (1mg/ml, 1:10) during 30 min at 25°C. The nanoparticles were then washed by centrifugation and resuspension in 0.1 M sodium phosphate buffer pH 8.0 three times.

#### Co-entrapment with magnetic nanoparticles

10 μL of a 25 mg/mL solution of MNPs (chemicell FluidMAG-PAA, 200 nm) were brought to a magnetic separation rack during 5 min. The supernatant was removed and resuspended in the same volume of 0.1 M sodium phosphate buffer, pH 8.0. The co-entrapment procedure was the same as the one described above for the entrapment of HRP but adding the washed 10 μL suspension before the TMOS addition.

Aliquots of 0.4 mL of oxidized horseradish peroxidase solutions (protein concentration 1 mg/mL) in potassium phosphate buffer (0.1 M, pH 8.0) were mixed with 0.1 mL of 10% polyethyleneimine (PEI) adjusted to pH 8.0 with HCl and 10 μL suspension of the washed magnetic nanoparticles. The mixture was incubated for 10 min under gentle agitation at 25°C after which 0.1 mL of a hydrolyzed TMOS solution prepared by diluting TMOS in hydrochloric acid (1 mM) to a final concentration of 1 M, were added.

The resultant entrapped HRP preparation containing MNPs (BioSi@HRP_MNP) was then centrifuged (13500 rpm) for 5 min and washed five times by centrifugation and resuspension in 0.6 mL of sodium phosphate buffer 0.1 M pH 8.0. The nanoparticles suspension was sonicated in an ultrasonic cleaner at 130 W and 20 kHz (SONICS & MATERIALS, INC.) for 5 min and finally reduced using the above-mentioned protocol for a three-dimensional covalent immobilization.

#### General procedures for nanoparticles characterization

The morphology and particle size distribution of the resulting nanoparticles (NPs) were characterized by Environmental Scanning Electron Microscopy (ESEM) images were obtained using a QUANTA-FEG 250 microscope in “wet-mode” using a Peltier stage and a gaseous secondary electron detector (GSED). The secondary electron images were taken at a voltage range between 10–15 keV, low temperature (1°C), high chamber relative humidity (100%) and high Pressure (659 Pa) to maintain the wet sample hydrated avoiding the sample damage during the observation. The sample was prepared in milliQ water in a dilution of (1:10000) and sonicated prior to measurements for 3 min to improve the dispersity.

Dynamic light scattering (DLS) and Z-potential measurements were performed on a Malvern ZS nano instrument at 25°C. Each sample was prepared by diluting the sample (1:100000) with milliQ water of which 1 mL was added to a quartz cuvette. They were measured 10 times, with a combination of 3 runs per measurement. The data was analysed using Zetasizer software. Similarly, the z-potential was measured using the same sample in a Folded Capillary Zeta Cell and the sample was measured 10 times and analysed using the aforementioned software.

Magnetic characterization was performed as follows: 50 μl of the liquid sample were placed inside a polycarbonate capsule and sealed with vacuum grease for their magnetic characterization. The magnetic characterization was performed in a Quantum Design (USA) MPMS-XL SQUID magnetometer. Field dependent magnetization was recorded at 300 K under decreasing field starting from 2 T, in the field range between -2 T and 2 T.

#### Reuse of nanohybrid by magnetic separation

The reusability of the immobilized enzyme nanohybrids was studied by repeated usage for 10 enzymatic cycles. Enzymatic reactions using 9.1 mM of ABTS and 0.3% hydrogen peroxide as substrates were performed in a 2 mL reaction volume containing potassium phosphate buffer pH 5.0, and a fixed amount of immobilized enzyme (20 U). Between each cycle, the nanohybrids were carefully separated using a magnetic separator (Chemicell- MagnetoPURE BIG SIZE) and then resuspended in the reaction mixture. The reactions were measures spectrophotometrically at 405 nm for 2 min. The activity determined during the first cycle was considered 100% for the calculation of remaining percentage activity after each use.

#### Determination of reducing sugars by DNS method

To analyse the concentration of trehalose by DNS, serial dilutions of trehalose from 0.07 g/L to 10 g/L were made. To 2 mL of these solutions 1 mL of dilute hydrochloric acid was added and boiled for 1 min following which was cooled and the acid was neutralized with sodium hydrogen carbonate. Similarly, the supernatant of an immobilized preparation (stored for 1 month), for which we intended to observe trehalose leakage, was hydrolysed to convert the non-reducing sugar into a reducing sugar.

To the hydrolysed preparations 250 μl of DNS reagent was added. The mixture was heated at 90ºC for 15 min to develop the range of colours which formed the standard to analyse our sample. Finally, distilled water was added to bring the final volume to 1 mL which were cooled to room temperature and the absorbance was recorded at 570 nm in a spectrophotometer.

#### Temperature profile of the nanoabiocatalysts

To study the optimum temperature, the reactants were heated in a water bath to a range of temperatures (20°C to 60°C). Upon reaching the desired temperature 10 μL enzyme was added to the reactant mixture and measured spectrophotometrically at 405 nm for 2 min.

To analyse the range of thermal stability, the enzyme preparations were incubated in 0.1 M sodium phosphate pH 8.0 for 1 h at the aforementioned temperatures (20 to 60°C) after which the activity was measured spectrophotometrically at 405 nm for 2 min.

#### pH stability analysis of the nanobiocatalysts

HRP preparations were incubated in 0.1 M sodium phosphate buffer pH 7.0 and 8.0, 25 mM sodium acetate pH 3.0, 4.0, and 5.0, and 25 mM sodium bicarbonate pH 10.0. Aliquots of soluble and entrapped suspensions were withdrawn, and their residual activity was measured as previously described after 1 h of incubation.

#### Thermal stability of the nanobiocatalysts

The thermal stability was carried out at 50ºC, wherein, aliquots of soluble and entrapped suspensions were withdrawn at different time intervals and their residual activity was measured as previously described. Residual activity was defined as:

$$Residual\;Activity=\frac{a}{a_{0}}$$ (1)

Where a are the IU at a time point and a_0_ is the initial activity in IU. Biocatalysts inactivation was modeled based on the deactivation theory proposed by Henley and Sadana[31] using Graph Pad Prism. Inactivation parameters were determined from the best-fit model of the experimental data which was the one based on two-stage series inactivation mechanism without residual activity, as represented in the following scheme:

$$E\overset{k_{1}}{\to }E_{1}\overset{k_{2}}{\to }E_{d}$$

where k_1_ and k_2_ are first-order transition rates constants. E, E_1_ and E_d_ are the corresponding enzyme species of progressively less specific activity, being the last one completely inactive. The mathematical model that represents this mechanism is:

$$\frac{a}{a_{0}}=\left(1+\alpha (\frac{k_{1}}{k_{2}-k_{1}})\right)e^{-k_{1}\times t}-\left(\alpha (\frac{k_{1}}{k_{2}-k_{1}})\right)e^{-k_{2}\times t}$$ (2)

where *α* is the enzyme specific activity. Inactivation parameters were determined from the best-fit model of the experimental data. Half-life (time at which the residual enzyme activity is half of its initial value; t_1/2_) was used to compare the stability of the different biocatalysts, being determined by interpolation from the respective model described by Eq 2. The stability factor (SF) was the parameter used for a quantitative comparison of the stability of the biocatalysts and was found by

$$Stabilization\;factor=\frac{t1/2}{{t1/2}_{0}}.$$ (3)

Where *t*1/2 is the half life time of the more stable sample and *t*1/2_0_ is the half life time of the less stable sample.

#### Oxidation of 3-IAA by enzymatic preparations

The oxidation of 3-indole acetic acid (3-IAA) by soluble and immobilized preparations (1 UI) was carried out as in [28], in 100 mM sodium acetate buffer pH 5.0 containing 500 mM of 3-IAA at 25°C for 2 h. An aliquot of reaction mixture was injected into a reverse-phase HPLC on a C18 Columbus column at 25°C using an isocratic elution buffer of methanol/1% acetic acid mixture (40:60, v/v) at a flow rate of 0.6 mL/min. The eluted products were monitored at absorbance of 250 nm using an Agilent 1100 series detector. The retention time for 3-IAA was 22 min and the reactive oxygen species were eluted from 3 min to 20 min.

#### Cytotoxicity and cell viability 3-(4,5-Dimethylthiazol-2-Yl)-2,5-Diphenyltetrazolium Bromide (MTT) assay

The experiments were carried out with human colorectal cancer (HCT 116) cell line. HCT116 shows less internalization compared to other cell lines and withstand higher temperatures[32] which suits future in vitro experiments related to the application of the nanobiocatalysts in direct enzyme prodrug activation. Additionally, it has been optimized for future 3D model experiments and genetically modified to constitutively expresse luciferase, which allows better biomedical imaging of tumour growth in in vivo experiments. To study the cytotoxic effect of the nanohybrids with 3-IAA we plated 15 x 10^3^ cells HCT 116 per well in a 96-well plate and incubated in DMEM with 10% FBS, 1% glutamine (Invitrogen) and 1% penicillin/streptomycin (Invitrogen) for 24 hours in an incubator at 37ºC and in presence of 5% CO_2_. We then removed the medium and washed the cells with PBS. Following which we added varied nanohybrids with varied enzymatic activity resulting in 0.5 IU, 1 IU or 2 IU along with the prodrug with final concentration in the well of 1 mM or 2 mM of 3-IAA. The medium selected for this incubation was PBS. The cells when then incubated for 6 h at 37°C. Different controls were prepared to analyse the cytotoxicity effect of every single component; nanohybrids and the prodrug. Besides, samples with the enzyme in suspension alone and in combination with the prodrug were prepared to be able to compare the efficiency of the nanohybrids. The supernatant was then removed and 100 ul of complete medium and 10 ul of MTT (5mg/ml) was added per well and was incubated for 2 h 37°C, until intracellular purple formazan crystals are visible under microscope. The plate was centrifuged at 1200 rpm for 25 min at RT. Following the removal of the supernatant and addition of 100 μl of DMSO per well to solubilize the formazan crystals, the absorbance was measured at 570 nm. Every sample was prepared in triplicates.

## Results and discussion

**Fig 1:**
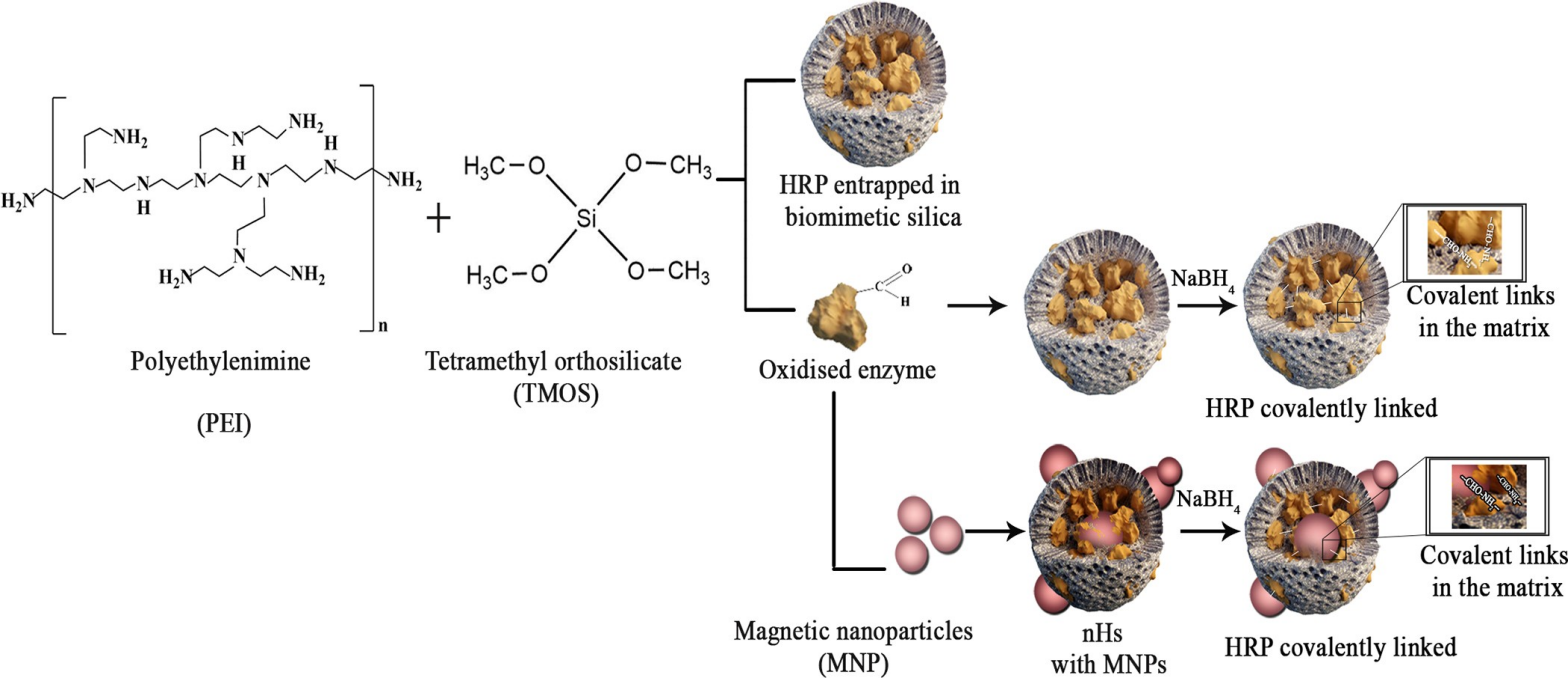
Schematic representation of different synthesis of silica and the co-entrapment of the enzyme with magnetic nanoparticles.

Sol-gel and functionalized mesoporous Si have been previously used for enzyme immobilization. These methods have some inherent limitations such as harsh synthetic conditions or poor retention of the immobilized enzyme different from biomimetic Si synthesis [19,33]. The immobilization approach follows a one-pot procedure, wherein, Si synthesis and enzyme entrapment occur simultaneously. Biomimetic reactions for Si deposition and HRP entrapment has been utilized in the past with modest results in terms of stabilization [28,34]. However, in previous reports we and others have demonstrated that this type of immobilization technique necessitates tailoring to the particular enzyme and bioconversion for optimal properties of the biocatalyst [13,35]. In order to maximize the stability and enzymatic performance of HRP immobilized preparations, we studied three different configurations of the immobilized enzyme Fig 1.

We first studied the entrapment process of HRP solely in biomimetic Si and the properties of the obtained immobilized preparation. A range of HRP concentrations were used to evaluate the immobilization parameters in silica entrapment and select a minimal amount of enzyme that allowed the preparation of a high specific activity biocatalyst S1 Table. In the light of the results obtained we chose 1 mg/mL HRP concentration for further experiments using different MW polyethyleneimine (PEI) as a catalyst for the Si deposition. PEIs with MW of ~ 1300, 2000, 25000 and 60000 were used to study the role of the amine rich catalyst in the nanoparticle synthesis and HRP stabilization. PEI is a polymer containing primary, secondary and tertiary amino groups, having a strong anion exchange capacity under a broad range of conditions, and the capability to chemically react with different chemical groups on either an enzyme or a support. Difference in PEI sizes could not only affect biological aspects of the biocatalyst but also its physicochemical properties [8,36]. Additionally, post immobilization chemical strategies have often improved otherwise unstable immobilized enzyme preparations [37,38]. We therefore attempted to crosslink the enzyme once entrapped within the Si matrix via chemical connection of aldehyde groups of the enzyme to unreacted amino groups of the PEI Fig 1. To the extent of our knowledge, there is no previous report on this approach for HRP immobilization in biomimetic Si nanoparticles (NPs). Considering the degree of glycosylation of this enzyme [39], we performed a standard mild oxidation via NaIO_4_ of the enzyme. This treatment generates aldehyde groups on HRP sugar residues that could form Schiff´s bases with amino groups of the PEI used as a catalyst for the Si deposition. Similar to the chemistry used to immobilize proteins on glyoxyl activated supports [40], a further reducing step via Na_2_BH_4_, would transform the first reversible interaction between the enzyme and the matrix into a three dimensional multiple covalent attachment of HRP within the Si particles Fig 1. The strategy was followed in the presence of trehalose, a common additive used to gain protein stability [12]. A direct correlation between the surface tension of trehalose solutions and the thermal stability of various proteins has been established and it is also known that trehalose significantly increases the half-life of HRP [5]. No impact was observed in the immobilization percentage (%I) and immobilization yield (%Y) of the preparations after including the trehalose and the reduction step in the preparation of the nanobiocatalyst S2 Table.

Regarding the effect of the different PEI MW, except for the BioSi@T_HRP_PEI_60000, the immobilization percentage (%I) and immobilization yield (%Y) of the different HRP were above 60%. Protein immobilization yield was between 78±2% and 83±4% for all the preparations. The nanohybrid obtained with PEI MW 1300 displayed the higher results for %I and %Y Table 1. This preparation contained 1.33 mg HRP/g of wet support and 1.05 IU/g of wet support.

**Table 1:** Immobilization parameters of nanohybrids with different sizes of PEI.

Entrapment	Immobilization (%)	Immobilization yield (%)	Protein immobilization yield (%)
BioSi@T_HRP_1300	60 ± 4	78 ± 1	82±1
BioSi@T_HRP_2000	61 ± 3	77 ± 2	80±2
BioSi@T_HRP_25000	64 ± 2	70 ±5	77±1
BioSi@T_HRP_60000	70 ± 2	33 ± 5	77±4
BioSi@T_HRP_MNP_1300	83 ± 5	71 ± 2	78±1
BioSi@T_HRP_MNP_2000	76 ± 2	69 ± 4	78±3
BioSi@T_HRP_MNP_25000	77 ± 2	70 ±5	83±4
BioSi@T_HRP_MNP_60000	69 ± 2	57 ± 5	78±2

None of the immobilized preparations obtained showed enzyme leakage as measured in the supernatant of a suspension incubated a 4°C for 1 month.

When adding MNPs to the synthetic mixture, we observed that for the nanohybrids with PEI MW 60000 there was an increase in the %Y from 33±5% to 57±5%. Probably, at higher PEI MWs a denser cover of Si could affect the partition of substrate/product through the solution thus yielding lower expressed activity of HRP after immobilization. The presence of MNPs could direct a Si deposition in a more compact polymeric shell, reducing mass transfer limitations.

**Fig 2:**
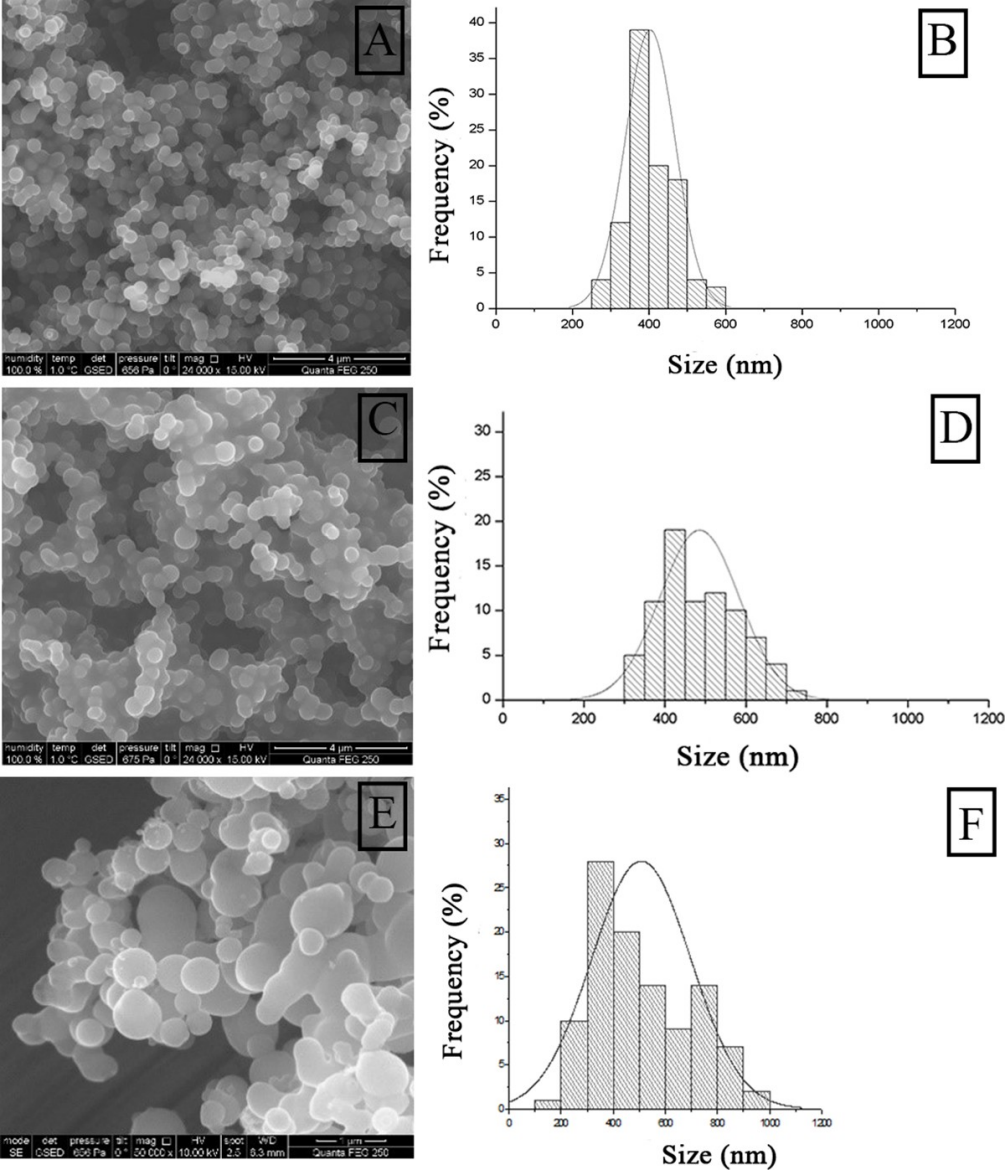
Analysis by scanning electron microscopy (SEM) of nanohybrids using PEI MW 1300. A) BioSi@HRP, C) BioSi@HRPox E) BioSi@T_HRP_1300. B, D y F) correspond to histograms of frequency of particles versus their particle size in each case.

Analysis by SEM showed that when entrapment of HRP using PEI MW 1300 was performed without chemical modification, biomimetic Si formed as preferentially disperse particles with a nanosized diameter range of ~ 300–550 nm with a sharp accumulation of ~ 400 nm diameter particles Fig 2A and 2B. When oxidized HRP was entrapped and the resulting particles submitted to NaBH_4_ reduction, biomimetic Si formed as interconnected randomly agglutinated particles of approximately ~ 300–800 nm Fig 2C and 2D. In this case, the Gaussian fitting of the nanoparticle size histogram showed a wider size distribution of the material, demonstrating an effect of the chemical modification of the enzyme on the synthesis of biomimetic silica. The oxidation of enzymatic sugar residues may change the ionization state of the enzyme at pH 8.0 which could alter the Si deposition process. Previous reports have already conferred a fundamental role of the interplay of attractive/repulsive electrostatic interactions during Si synthesis on the particle size and distribution of the material [22,41]. The presence of trehalose during Si synthesis also affected the size distribution of the particles obtained with diameters ranging from 100 to 1000 nm. Moreover, trehalose significantly impacted the homogeneity of the sample Fig 2E and 2F. Given that the amount of protein used in all the entrapment experiments was the same (1 mg/mL), size dispersion can be attributed solely to trehalose. These results corroborate with Rodriguez et al [42] that found that the addition of carbohydrates to standard hydrostatic solutions altered the size of the spherical Si particles obtained from *in vitro* polycationic peptide-mediated biosilicification. Although their findings were obtained after Si precipitation without protein in the synthetic mixture, it became clear that sugar molecules imparted some degree of morphological control on the deposited silica.

**Fig 3:**
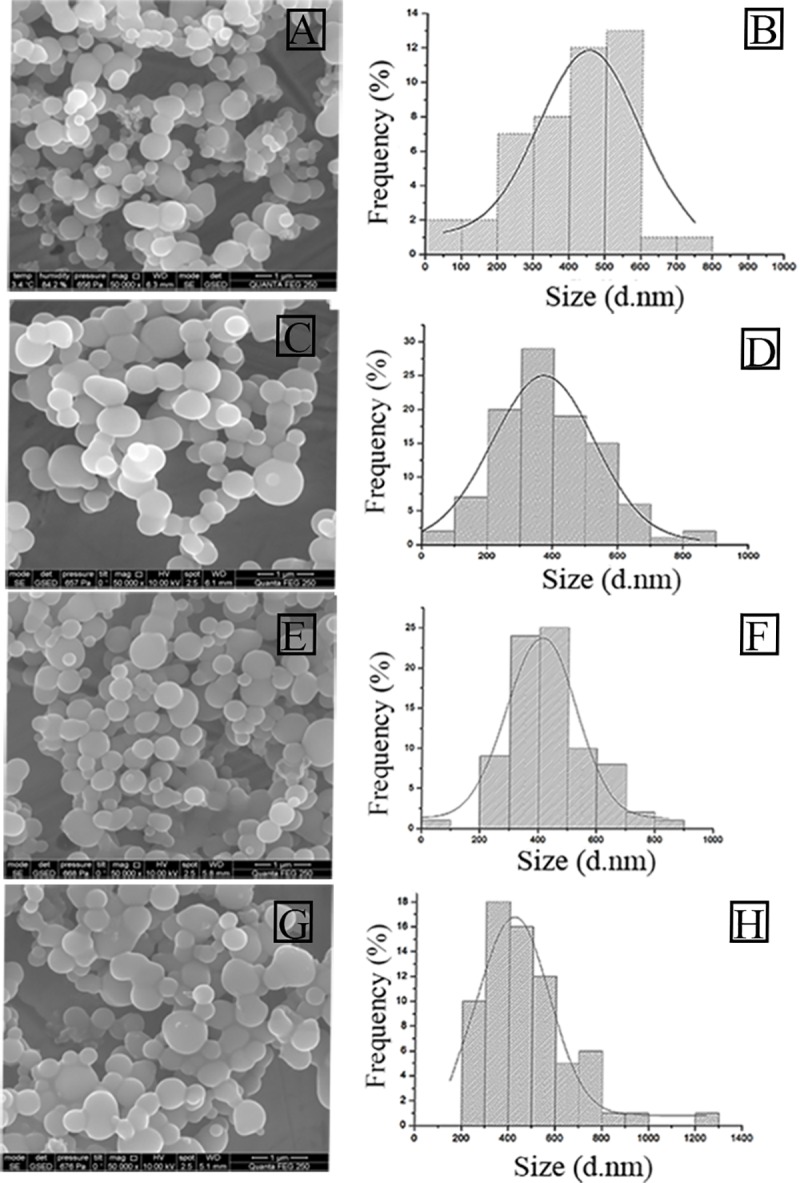
Analysis by SEM of nanohybrids with distinct PEIS. A) BioSi@T_HRP_MNP_1300. C) BioSi@T_HRP_MNP_2000, E) BioSi@T_HRP_MNP_25000 and G) BioSi@T_HRP_MNP_60000. Similarly, B, D, F, H correspond to their histograms analyzed using ImageJ and Origin 8 Pro.

Table 2 shows the results for DLS analysis of the different nanohybrids with and without MNPs. Addition of MNP in the synthetic mixture provided nanohybrids with smaller hydrodynamic sizes making the final diameter of the hybrid independent of the size of the PEI used (~500–600 nm). This correlates with the results obtained for an increase in %Y after addition of MNPs and the analysis by SEM of the samples that included MNPs Fig 3. The samples that included MNPs showed again interconnected particles of a mean diameter of 400–450 nm Fig 3. The particle size distribution has a high dispersity that correlates with increased PDI results Table 2 that did not impact on the activity of the nanobiocatalysts.

**Table 2:** Dynamic light scattering and net charge analysis of nanohybrids.

Hybrids	Hydrodynamic size (nm)		Poly dispersity index (PDI)		Zeta potential (mV)	
	MNP (-)	MNP (+)	MNP (-)	MNP (+)	MNP (-)	MNP (+)
**BioSi@THRP_1300**	630 ± 26	684± 68	0.199±0.136	0.311±0.03	6.79±0.791	23.4±4.68
**BioSi@THRP_2000**	815 ± 52	589 ± 28	0.104±0.067	0.321±0.044	6.87±0.701	21.5±4.24
**BioSi@THRP_25000**	535 ± 23	491 ±26	0.129±0.063	0.402±0.080	8.31±0.701	11.6±5.46
**BioSi@T_HRP_60000**	1026 ± 83	543 ± 21	0.207±0.089	0.354±0.059	9.81±1.12	15.5±4.00

**Fig 4:**
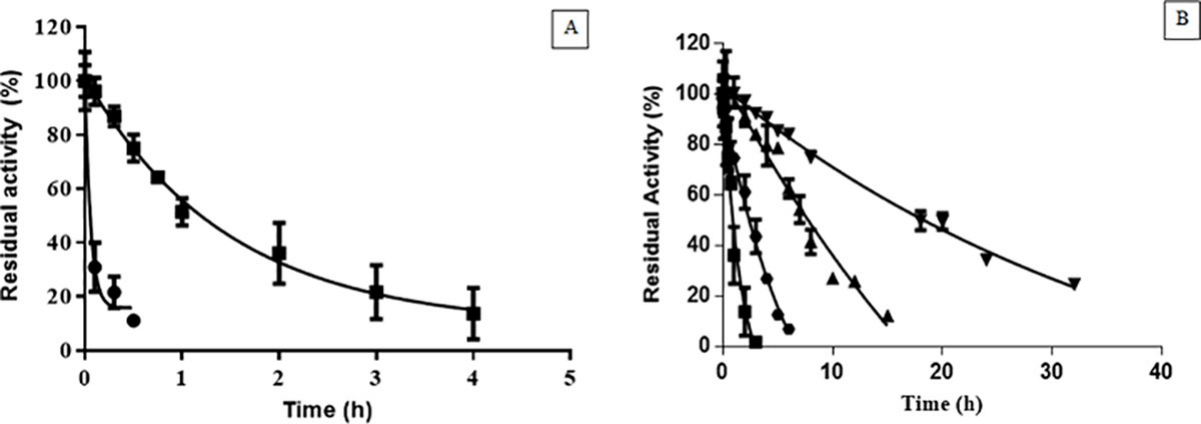
Thermal stability at 50°C of different HRP preparations. **A)** Soluble enzyme (HRP) (●), BioSi@HRP (■). Aliquots were taken at mentioned time intervals and measured spectrophometrically at 405nm. The half-lives were determined as 0.04 h and 1.09 h for the soluble and immobilized preparations, respectively. **B)** Thermal stability of the BioSi@HRP (■), BioSi@HRPox (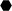), BioSi@T_HRP_1300(▲) and BioSi@T_HRP_MNP_1300 (▼) showing half-lives 1.09 h, 2.5 h, 7.02 h, 21.3 h.

Stabilization of enzymes is a key factor to determine their full potential as biocatalysts. Our studies on thermal stability of the entrapped enzyme demonstrated that, after fitting the experimental data to the exponential model from Henley and Sadana [43], the physically entrapped HRP (BioSi@HRP) had a half-life time of 65.4 min at 50°C compared to the soluble enzyme that reached 50% of its initial activity after only 2.4 min Fig 4. Thermal stabilization improved considerably after chemical modification of the nanobiocatalysts Fig 4B. When using PEI MW 1300, modified nanoparticles showed a half-life time of 150 min compared to 65.4 min of the unmodified entrapped HRP. The effect of trehalose and the chemical crosslinking on the thermal stability of the immobilized HRP was additive, as the preparation had a stabilization factor (SF) of 176 compared to the soluble HRP Fig 4B. Since the Si NP is a porous material, we excluded the possibility of trehalose leakage by incubating an immobilized preparation in suspension at 4°C for 1 month and determination of reducing sugars in the supernatant. On measurement of the supernatant, no trehalose was detected under these conditions S3 Table. Moreover, a suspension of BioSi@T_HRP_MNP_1300 containing 12.5 IU/mL in sodium phosphate buffer 0.1 M pH 8.0, retained 84%±3 (10.5±0.4 IU/mL) of its initial activity after 6 months of shelf storage at 4°C.

We believe these results demonstrate that a three-dimensional rigidification of the enzyme structure is a determinant factor to a drastic improvement in its stability. Some indications of this effect had been previously obtained by immobilization of enzymes on matrixes modified with polymeric molecules in which it was believed that regions of the biomolecules were embedded within the support, improving their stability [38]. However, an entrapment process assures that most of the enzymatic molecules lay within the matrix which is fundamental to reinforce our three-dimensional stabilization hypothesis.

When stability of nanohybrids including MNPs was studied at 50°C, we observed a 532 SF of the enzyme entrapped in Si with MNPs (BioSi@THRP_MNP_1300) compared to the soluble enzyme Fig 4B.

Considering that the MNPs contain primary amino groups that could further react with the aldehyde generated upon mild oxidation in the HRP, we believe the presence of the MNPs provided an additional source of functional groups for multi-point covalent interaction. Moreover, the MNPs offer a more rigid surface to the enzyme to the flexible Si network formed as a shell of the nanohybrid. This may restrain enzyme distortion and contribute to a greater stabilization.

**Fig 5:**
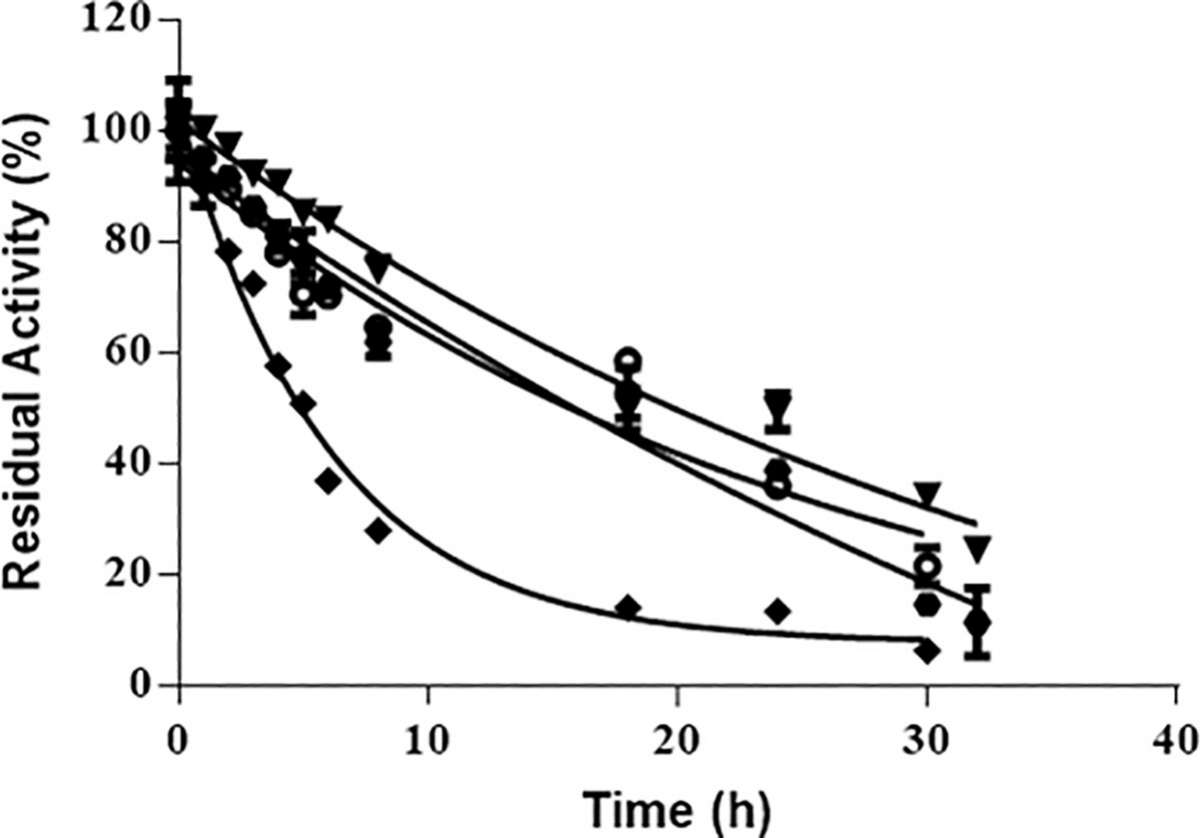
Thermal stability of the enzyme preparations with distinct polyethyleneimines (PEI). (BioSi@T_HRP_MNP_1300 (▼), BioSi@T_HRP_MNP_2000 (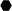), BioSi@T_HRP_MNP_25 000 (♦), BioSi@T_HRP_MNP_60 0000 (o) entrapped in silica with a magnetic core. The half-lives were determined as 21 h, 20 h, 8 h, 22 h, respectively.

The nanohybrids with distinct PEIs showed an increase in the half-life similar to nanohybrids with PEI MW 1300 respect to the soluble with an exception of PEI MW 25000 which showed a SF of 20 with respect to the soluble enzyme Fig 5. The branched nature of this PEI amplifies the loading enzyme but could leave the enzyme more exposed to degradation caused by temperature increase [44].

Reports for immobilization and stabilization of HRP are ubiquitous. The majority of these reports include inhouse fabricated supports and conditions for stability evaluation vary extensively. For instance, using commercially available biogenic porous silica, Sahare et al found a SF of 23[45] with an immobilized preparation with similar specific activity to the one used in our work. HRP immobilized onto PVA–PAAm nanofibers was found to retain 64% of its initial activity at 4°C after 55 days which represents a lower storage stability compared to the nanohybrid prepared herein[46]. For each of these examples it is important to highlight that the nature of the support used and the chemistry of HRP immobilization is different for that developed in this work. The different immobilization strategies might better suit precise applications, which eventually will make the stability results relevant.

We have selected BioSi@THRP_MNP_PEI_1300 for further experiments, under the premise that it was the most thermostable preparation obtained in our work with higher %Y and %I. We have studied the remaining activities of HRP preparations after 1 hour of incubation in several pHs S1 Fig. pH stability often restrains the applicability of enzymatic preparations as it may have a profound impact on the loss of structural integrity of many proteins. Our results demonstrate that the nanohybrids were more stable under acidic pH. There was no observed effect of the MNPS on the pH stability. Additionally, no loss of integrity of the NPs was observed after 1 hour of incubation at the different pHs.

Optimal pH and optimal temperature of HRP did not change upon integration of the enzyme in the nanohybrids S2A and S2B Fig.

**Fig 6:**
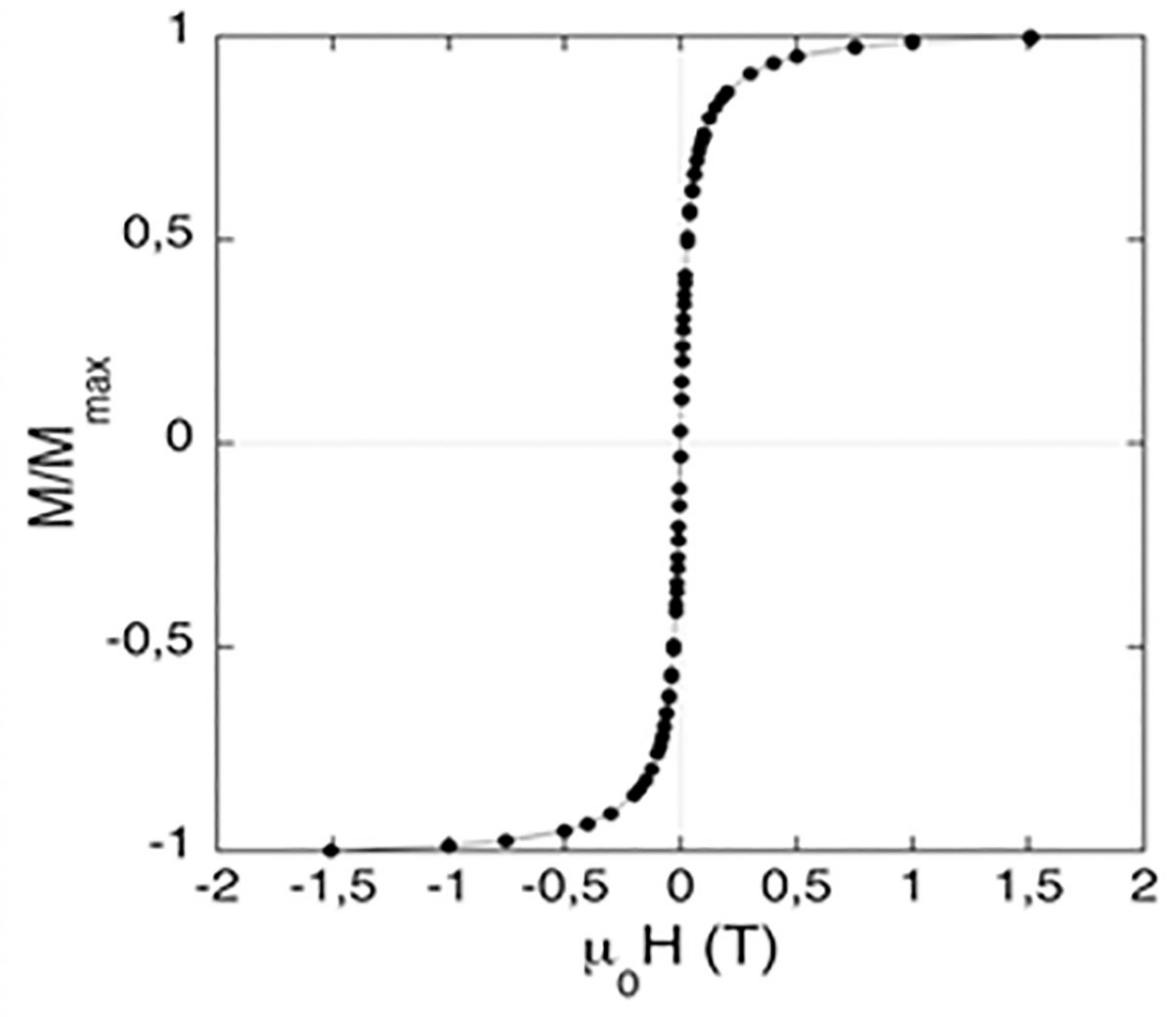
Field dependent magnetization of BioSi@T_HRP_MNP_1300. The measurements are shown at 300 K after diamagnetic correction.

It was important to demonstrate that after the Si modification, the nanohybrids maintained their superparamagnetic properties. Fig 6 shows the field dependent magnetization of the BioSi@THRP_MNP_1300 at 300 K. The sample displayed superparamagnetic behaviour with negligible coercivity at zero field.

**Fig 7:**
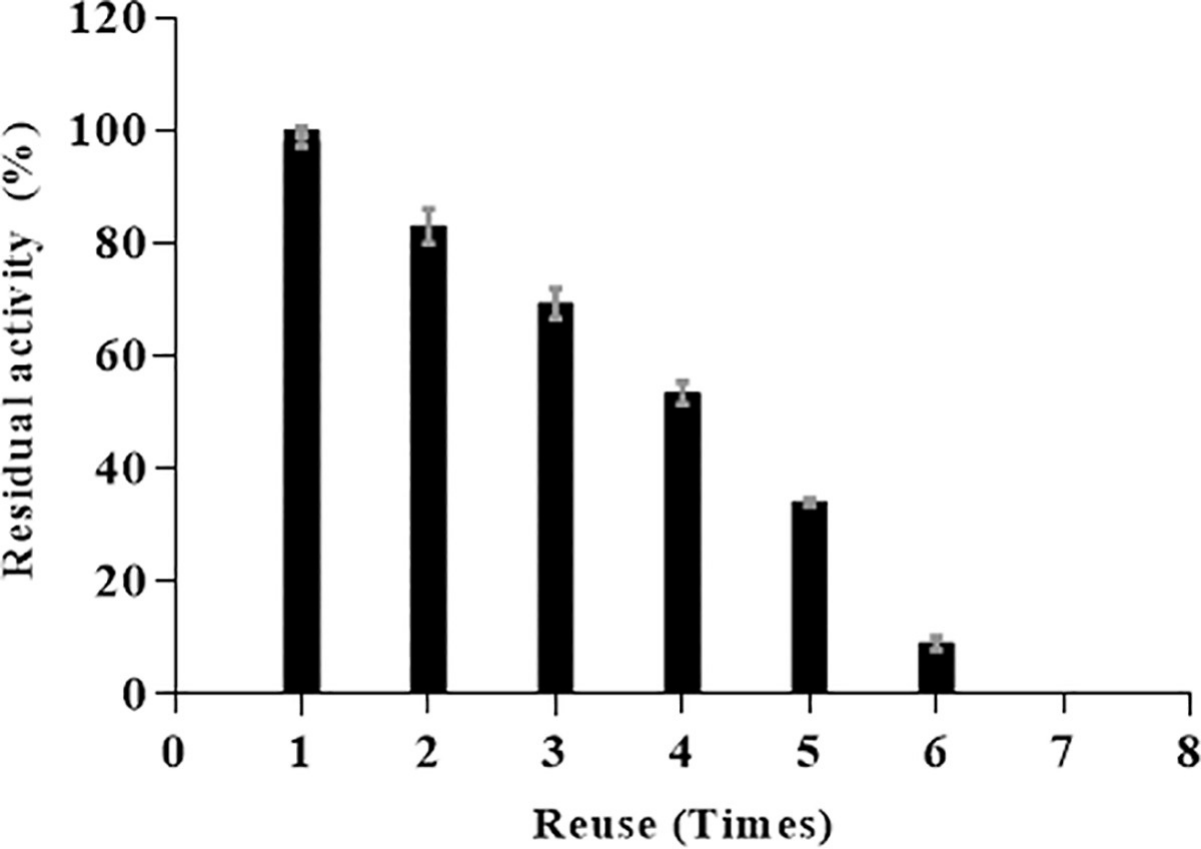
Operational stability of BioSi@T_HRP_MNP_1300. Residual enzyme activity after 6 reuses with substrates (ABTS and H_2_O_2_) and separation using a magnetic separator.

Operational stability of the BioSi@THRP_MNP_1300 was also assessed after several enzymatic cycles using the chromogenic substrate ABTS and hydrogen peroxide. In all studied cycles, the immobilized enzyme was magnetically separated and was assessed for its remnant catalytic activity. After 5 reuses the nanohybrids maintained 30% of its initial activity Fig 7.

**Fig 8:**
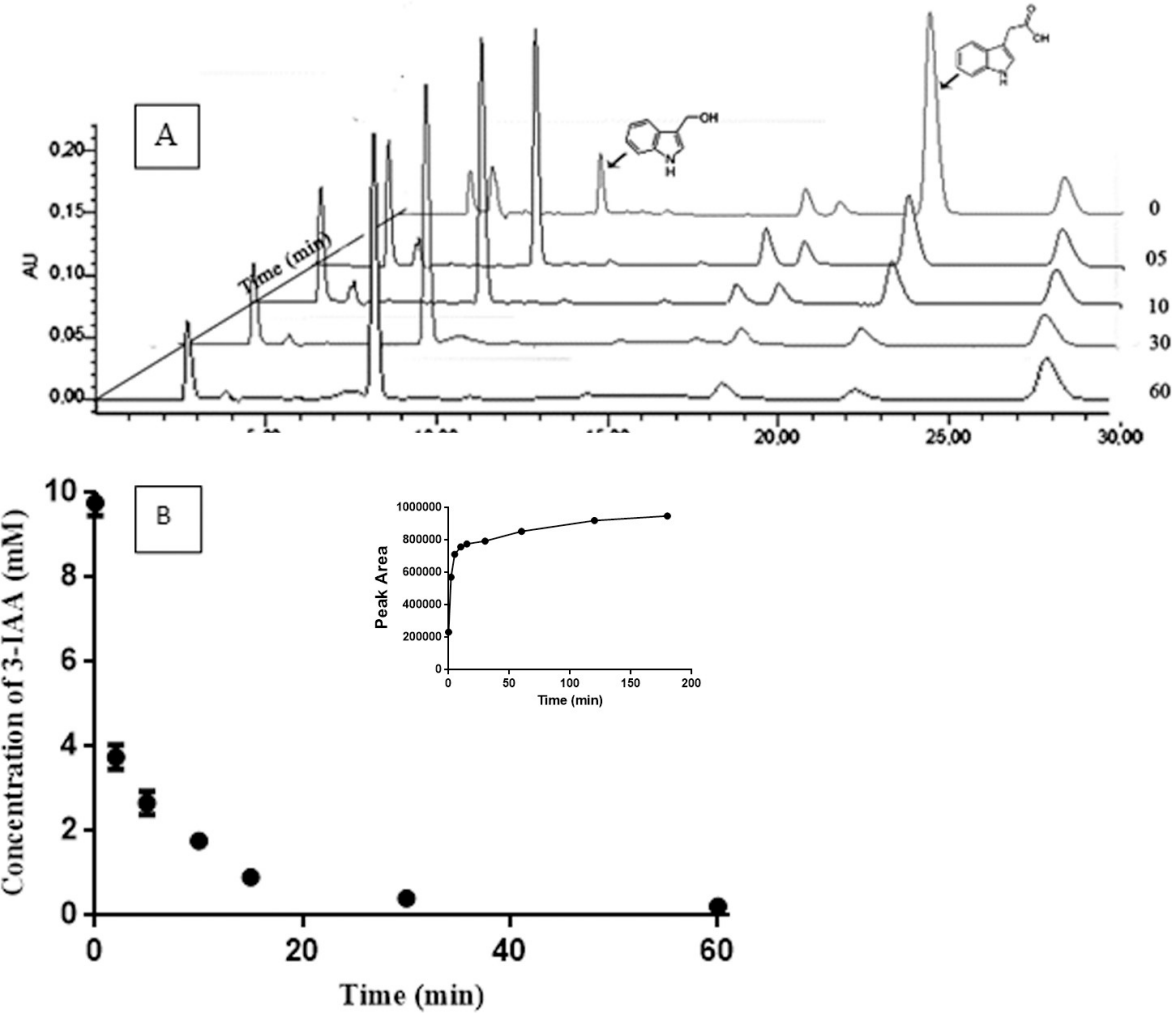
Oxidation of 3-IAA by the nanohybrids. A) Conversion of 3-IAA by nanohybrids BioSi@T_HRP_MNP_1300 at different intervals of time B) Conversion kinetic of 3-IAA by the nanohybrids. Reactions were carried at 25°C using 1 UI in a 100 mM sodium acetate buffer at pH 5.0 containing 500 mM of 3-IAA. Further details are described in Methods. Inset- Increment in the concentration (area) of the radical 3-ox-indol-carbinol.

As a proof of its utility in a biotechnological relevant biotransformation, we studied the oxidation of 3-IAA. This non-toxic plant hormone has been examined as a prodrug candidate as, upon transformation to its oxidized species, it induces cellular apoptosis in cancerous lines. HRP has been proposed as oxidizing enzyme of this compound for the so-called direct enzyme prodrug therapy. The biocatalytic performance of the BioSi@THRP_MNP_1300 was tested in batch conversion of 3-IAA into its oxidized species. HPLC elution profiles showed that the nanohybrids catalysed the complete oxidation of a 500 mM prodrug solution within 30 min of reaction with the generation of at least five oxidized products Fig 8A and 8B.

The major product is expected to be oxindol-3-yl carbinol for its distinctive spectra and matching retention time from previous works using the same HPLC analysis conditions. This type of immobilized biocatalyst could be potentially applied to biotransformations such us *in situ* clean-up of contaminated environments [47,48], lignin polymerization for hydrophobicity enhancement of fibres [49] or other polymerization reactions applied in pharmaceuticals [50].

**Fig 9:**
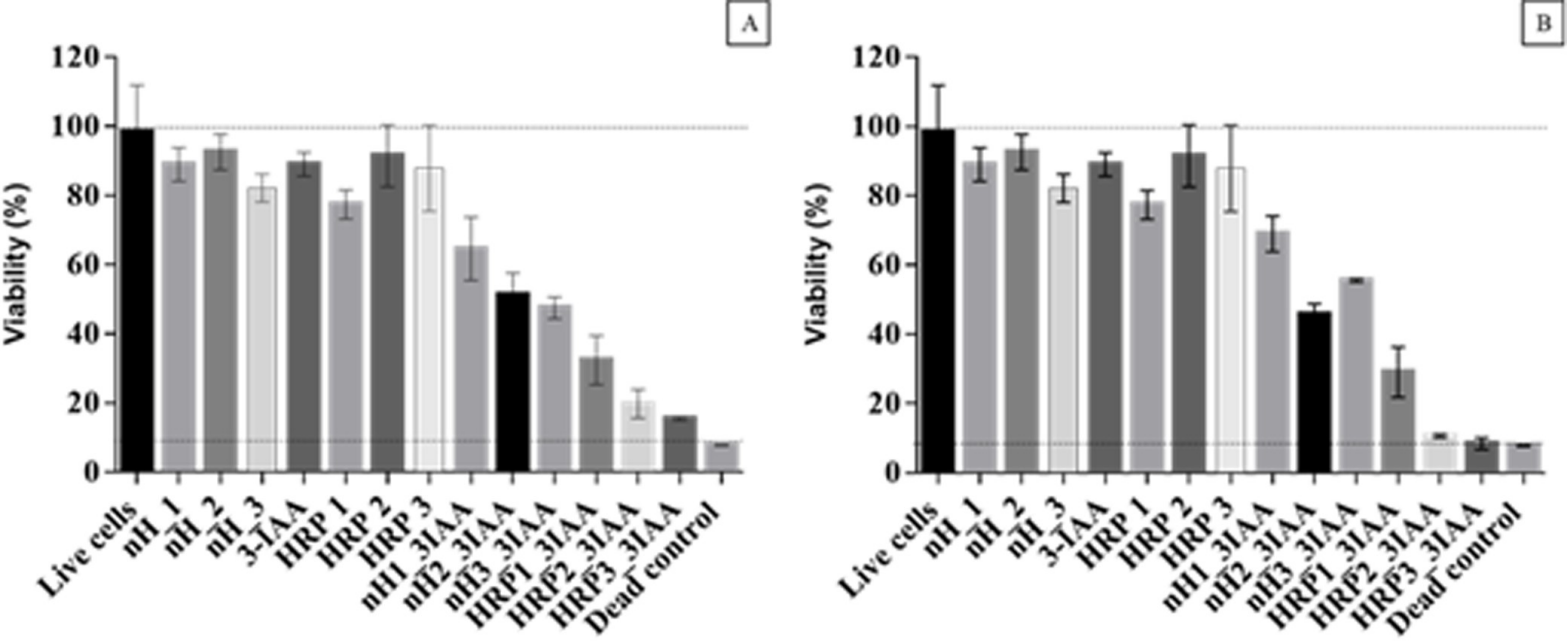
Effect of nanohybrids (BioSi@T_HRP_1300_MNP) on HCT 116 (ATCC) cell viability. Cytotoxicity studies using MTT were assayed. Cells were incubated for 6 h in PBS with concentrations of nanohybrids (0.5, 1 and 2 IU of Enzyme) and concentrations of prodrug 1 mM and 2mM. A) Corresponds to the data normalized against live control (cells with DMEM 10% FBS, considered as 100%) for an assay using 1 mM of 3-IAA. B) Corresponds to the data normalized against live control (cells with DMEM 10% FBS, considered as 100%) for an assay using 2 mM of 3-IAA Results were expressed as the mean +/- SD of triplicates of a representative experiment. Live cells: Control of live cells, nH_1/2/3: control of nanohybrid at 5, 10, 20 ug/mL containing 0.5, 1 and 2 IU/mL respectively, 3-IAA: Control of prodrug with concentrations of 1 and 2 mM, HRP1/2/3 ctrl: control with soluble enzyme at 0.5, 1 and 2 IU/mL; nH_1/2/3_3IAA: nanohybrids and prodrug combinations; HRP 1/2/3/_3IAA: reaction mixture with soluble enzyme and prodrug combinations.

Anticipating a possible biomedical application of the HRP entrapped nanomaterial produced herein, we further investigated its cytotoxicity towards the model colorectal cancer cell line HCT 116 (ATCC) (Fig 9A and 9B). NPs may cause adverse health effects resulting from damage to membranes, changes in protein folding, DNA mutation, blood abnormalities and oxidative stress injuries. Measurements of cell viability and proliferation can provide an indication of the safety of nanomaterials.

The results show that after 6 hours in PBS, BioSi@THRP_MNP_PEI_1300 is well tolerated by the cells as not more than ~15% growth inhibition is observed in a range of concentrations of 5, 10 and 20 μg/mL. Besides, when incubating the cells solely with the free enzyme or with the prodrug no cytotoxicity effect was observed. However, cell death was observed when 3-IAA and nanohybrids were co-incubated with the cells. Increasing amounts of enzyme units (0.5–2 IU/mL) in the assay showed a correlated decrease in cell viability demonstrating that the optimized nanohybrid is efficient in the generation of toxic radicals. We can also see in the results that the two prodrug concentrations selected resulted in very similar cell viability values.

It is worth noting that the soluble enzyme showed a greater cytotoxic effect in the presence of the prodrug in comparison to the immobilized one, as it is free and easily available for the oxidation of the prodrug. The rate of conversion is slower in the nanohybrids as the substrate had to traverse through the Si matrix to access the enzyme entrapped thus decreasing the rapid conversion of the radicals in the assay. However, we have demonstrated that integration of the enzyme in the composite material provided advantageous properties that counterbalance any decrease in the conversion rate of the prodrug 3-IAA.

Although extensive analysis is necessary to fully understand nanomaterials toxic effects, the results of MTT in this work confirmed the utility of BioSi@THRP_MNP_PEI_1300 in directed enzyme prodrug therapy (DEPT), which encourages further investigations into *in vitro* and *in vivo* effects of the material.

## Conclusions

In this work we have prepared a new nanosized hybrid material that combined MNPs, biomimetic silica and the enzyme HRP. The diameter and polydispersity of the *in situ* prepared nanoparticles demonstrated a dependence on the size of the aminated polymer PEI used to deposit the siliceous material and the addition of the magnetic nanoparticles during synthesis. Addition of the disaccharide trehalose and a post immobilization chemical modification of the organic/inorganic material provided exceptional stability to the enzyme without compromising its activity. In fact, the immobilized enzyme showed a significantly higher thermal stabilization factor compared with previous reports for HRP [51,52]. The superparamagnetic properties of the nanohybrid facilitated its separation in repeated batch transformations of a synthetic substrate. Our findings demonstrate that the material is not cytotoxic while it enabled the cytotoxicity of cancerous cells upon transformation of the prodrug 3-IAA. In summary, the unprecedented approach for the preparation of a nanohybrid biocatalyst provided excellent properties that could enable a range of potential applications. Further experiments on conversion of alternate substrates of immobilized HRP will broaden the range of applications of the system. Moreover, the cytotoxic studies carried out with the nanohybrid prepared herein encourages additional experimentation for a better insight into its biomedical potential.

## Supporting information

S1 Table Entrapment of different concentrations of soluble HRP in biomimetic silica nanoparticles. (DOCX)

S2 Table Immobilization parameters of nHs with different immobilization strategies. (DOCX)

S3 Table Determination of Trehalose via DNS assay. (DOCX)

S1 Fig pH stability of different HRP preparations. pH stability of the enzyme preparations: soluble enzyme (black), BioSi@HRPox (gray), BioSi@T_HRP_MNP_1300 (white).
(TIF)

S2 Fig Temperature profile of different HRP preparations. A) Optimal temperature of the enzyme preparations: soluble enzyme (black), BioSi@HRPox (gray), BioSi@T_HRP_MNP_1300 (white). B) Thermal stability of the enzyme preparations: soluble enzyme (black), BioSi@HRPox (gray), BioSi@T_HRP_MNP_1300 (white).
(TIF)
