# Supplementary material for: Design of stable magnetic hybrid nanoparticles of Si-entrapped HRP
Source: PLoS One. 2019 Apr 1;14(4):e0214004. doi: 10.1371/journal.pone.0214004 (PMC6443235; doi:10.1371/journal.pone.0214004)
Supplement: S1 Table — (DOCX) [file pone.0214004.s001.docx]

S**1 Table.** **Entrapment of different concentrations of soluble HRP in biomimetic silica nanoparticles.**

| Enzyme (mg/ml) | Immobilization  (%) | Immobilization yield (%) | IU  (IU/mg) | Protein immobilization yield (mg/mL) |
| --- | --- | --- | --- | --- |
| 0.5 | 94 ± 3 | 50 ± 2 | 1.042 ± 4E-03 | 0.30 |
| 1 | 85 ± 2 | 59 ± 2 | 1.025 ± 2E-03 | 0.38 |
| 3 | 83 ± 2 | 53 ± 3 | 1.286 ± 4E-03 | 0.39 |
| 5 | 82 ± 4 | 49 ± 6 | 1.700 ± 7E-03 | 0.31 |
| 7.5 | 63 ± 4 | 42 ± 2 | 2.125 ± 5E-03 | 0.20 |
| 10 | 54 ± 3 | 12 ± 3 | 1.887 ± 8E-03 | 0.02 |
| 20 | 34 ± 5 | 10 ± 5 | 1.900 ± 7E-03 | 0.02 |
