# Supplementary material for: Design of stable magnetic hybrid nanoparticles of Si-entrapped HRP
Source: PLoS One. 2019 Apr 1;14(4):e0214004. doi: 10.1371/journal.pone.0214004 (PMC6443235; doi:10.1371/journal.pone.0214004)
Supplement: S2 Table — (DOCX) [file pone.0214004.s002.docx]

**S2 Table**. **Immobilization parameters of nHs with different immobilization strategies.**

| **Entrapment (1 mg/ml)** | **Immobilization (%)** | **Immobilization yield (%)** |
| --- | --- | --- |
| BioSi@HRP* | 85 ± 2 | 59 ±2 |
| BioSi@HRPox-red | 73 ± 5 | 53 ± 5 |
| BioSi@T_HRP | 86 ± 2 | 43 ± 2 |
| BioSi@T_HRPox | 80 ± 3 | 62 ± 3 |
