# Supplementary material for: Design of stable magnetic hybrid nanoparticles of Si-entrapped HRP
Source: PLoS One. 2019 Apr 1;14(4):e0214004. doi: 10.1371/journal.pone.0214004 (PMC6443235; doi:10.1371/journal.pone.0214004)
Supplement: S3 Table — (DOCX) [file pone.0214004.s003.docx]

**S3 Table. Determination of Trehalose via DNS assay.**

| **Trehalose concentration (mg/mL)** | **Absorbance at 570 nm** |
| --- | --- |
| 0,07 | 0.035 |
| 0,15 | 0.048 |
| 0,31 | 0.051 |
| 0,62 | 0054 |
| 1,25 | 0.063 |
| 2,5 | 0.076 |
| 5 | 0.084 |
| 10 | 0.184 |
| Supernatant of sample analysed | 0.001 |
