# Supplementary figures and images for: Design of stable magnetic hybrid nanoparticles of Si-entrapped HRP
Source: PLoS One. 2019 Apr 1;14(4):e0214004. doi: 10.1371/journal.pone.0214004 (PMC6443235; doi:10.1371/journal.pone.0214004)

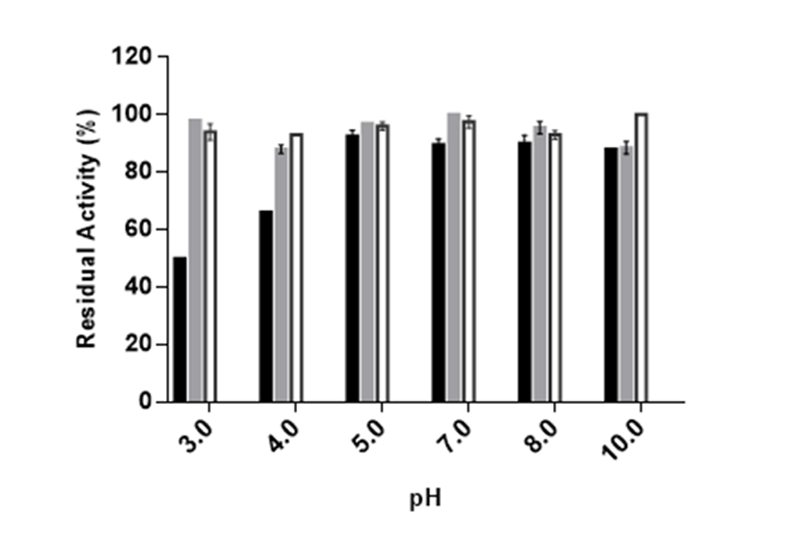

Supplement: S1 Fig — pH stability of the enzyme preparations: soluble enzyme (black), BioSi@HRPox (gray), BioSi@T_HRP_MNP_1300 (white). (TIF) [file pone.0214004.s004.tif]

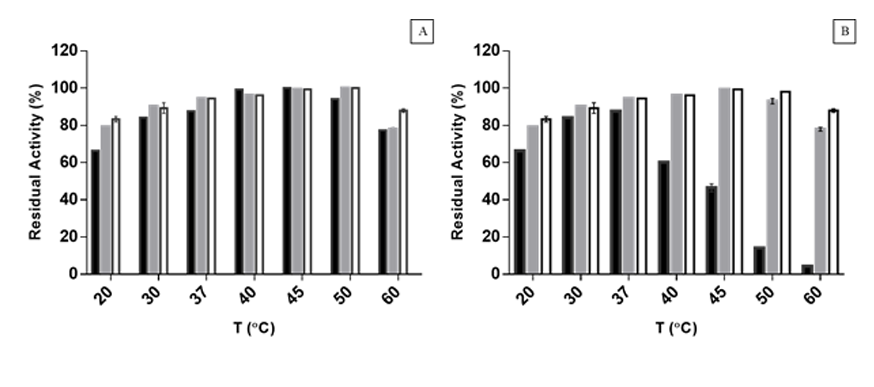

Supplement: S2 Fig — A) Optimal temperature of the enzyme preparations: soluble enzyme (black), BioSi@HRPox (gray), BioSi@T_HRP_MNP_1300 (white). B) Thermal stability of the enzyme preparations: soluble enzyme (black), BioSi@HRPox (gray), BioSi@T_HRP_MNP_1300 (white). (TIF) [file pone.0214004.s005.tif]
